# Critical Functions of PP2A-Like Protein Phosphotases in Regulating Meiotic Progression

**DOI:** 10.3389/fcell.2021.638559

**Published:** 2021-02-25

**Authors:** Wen-Long Lei, Wei-Ping Qian, Qing-Yuan Sun

**Affiliations:** ^1^Department of Reproductive Medicine, Peking University Shenzhen Hospital, Shenzhen, China; ^2^Fertility Preservation Lab, Reproductive Medicine Center, Guangdong Second Provincial General Hospital, Guangzhou, China

**Keywords:** protein phosphorylation, meiosis, PP2A, PP4, PP6

## Abstract

Meiosis is essential to the continuity of life in sexually-reproducing organisms through the formation of haploid gametes. Unlike somatic cells, the germ cells undergo two successive rounds of meiotic divisions after a single cycle of DNA replication, resulting in the decrease in ploidy. In humans, errors in meiotic progression can cause infertility and birth defects. Post-translational modifications, such as phosphorylation, ubiquitylation and sumoylation have emerged as important regulatory events in meiosis. There are dynamic equilibrium of protein phosphorylation and protein dephosphorylation in meiotic cell cycle process, regulated by a conservative series of protein kinases and protein phosphatases. Among these protein phosphatases, PP2A, PP4, and PP6 constitute the PP2A-like subfamily within the serine/threonine protein phosphatase family. Herein, we review recent discoveries and explore the role of PP2A-like protein phosphatases during meiotic progression.

## Introduction

In eukaryotes, reversible phosphorylation and dephosphorylation of proteins represents an prominent type of post-translational modification that has an crucial effect on controlling some cellular processes and events (Hunter, 1995). The state of protein phosphorylation can be adjusted by some highly conserved protein kinases and protein phosphatases (Mumby and Walter, 1993). In general, it is necessary for a number of critical biological events to keep a proper balance between protein kinases and protein phosphatases (Cassimeris, 1999). Disruption of this equilibrium can contribute to many pathological circumstances and even diseases. This spatial and temporal regulation of protein phosphorylation occurs not only in mitotic program, but also in meiotic progression.

Meiosis is a peculiar type of division in which one single round of DNA replication is followed by two sequential rounds of chromosome segregation (meiosis I and meiosis II), which is an important procedure for gamogenesis. Through this progression, diploid parent cells give rise to haploid gametes with the correct number of chromosomes. Similar to mitosis, meiotic division occurs in all eukaryotes and is an intricate event that is needed to change the cell cycle (Wolgemuth and Roberts, 2010). DNA replication and chromosome segregation both occur in meiosis. However, there are some other particular events in meiosis, such as homologous chromosome pairing, synaptonemal complex formation, double-strand break (DSB) repairing, meiotic recombination and a reductional division (Berchowitz and Copenhaver, 2010). Prior to the meiotic divisions, changing maternal and paternal chromosome behaviors including pairing, synapsis, and recombination must occur in a highly adjusted manner during prophase (Sato-Carlton et al., 2014). Therefore, according to the different behaviors of chromosomes, the prophase I also can be divided into five stages which are named as leptotene, zygotene, pachytene, diplotene, diakinesis. These events result in the mutual exchange of DNA material between homologous chromosomes and increasing genetic diversity (Bishop and Zickler, 2004). During meiosis I, homologous chromosomes are segregated whereas sister chromatids are still interacted on each other. Then sister chromatids are fully segregated in the second meiotic division (Canela et al., 2003; Qi et al., 2013). Errors in any of these events attribute to failure of the gametogenesis. In this progress, human oocytes have an abnormally high chromosome error rate that significantly increases with age, with severe results for human fertility (Keating et al., 2020).

A battery of protein phosphorylation and protein dephosphorylation events, which are adjusted by protein kinases and protein phosphatases, are critical for meiotic process (Bornslaeger et al., 1986; Lu et al., 2001). Protein kinases shift a phosphoryl group from adenosine triphosphate (ATP) to the hydroxyl group of serine, threonine and tyrosine residues, while protein phosphatases dephosphorylate protein by phosphate group hydrolysis and thus oppose the actions of protein kinases (Lillo et al., 2014). Among the phosphorylation, almost 98% of protein phosphorylation occurs on serine and threonine residues (Pearlman et al., 2011; Hunter, 2014). In human genome, there are more than 500 protein kinases encoded that catalyze the phosphorylation (Subramani et al., 2013). These protein kinases can phosphorylate the specific sites of target proteins. Nevertheless, it is insufficient for protein kinases alone to control dynamic processes. Because the phosphorylation of serine and threonine sites is extraordinary stable, which has long half-life (Lad et al., 2003), protein phosphatases can ensure that protein phosphorylation is dynamic and reversible (Nilsson, 2019). For various reasons, compared with the rich knowledge on protein kinases, there is a relative lack of information about the functions of protein phosphatases (Afshar et al., 2016). Among these conserved phosphoprotein phosphatases, PP2A, PP4, and PP6 constitute the PP2A-like subfamily within the serine and threonine protein phosphatase family (Bielinski and Mumby, 2007). These phosphoprotein phosphatases play crucial roles in multiple series of fundamental cellular events. Recent studies have implicated that PP2A-like protein phosphatases play critical roles in regulating meiosis. In this review, we will summarize recent discoveries and explore the role of PP2A-like protein phosphatases during meiotic progression.

### Classification of Protein Phosphatases

In the past decades, there are numerous studies about the biological roles of protein phosphatases, especially in meiosis. Generally, eukaryotic protein phosphatases can be divided into four major gene families based on specific substrate, catalytic activity and inhibitor sensitivity (Lillo et al., 2014). These families are phosphoprotein phosphatases (PPP), Mg^2+^/Mn^2+^-dependent protein phosphatases (PPM), aspartate-based protein phosphatases, and phosphotyrosine phosphatases (PTP) (Kerk et al., 2008). Among these families, the PPPs are the most comparatively conservative members across the whole eukaryotic species from yeast to human, indicating their “housekeeping” importance (Brautigan, 2013). In eukaryotic cells, almost 80% of the protein phosphatase activity is regulated by PPP family (Janssens and Goris, 2001). The PPP family includes seven members, namely PP1, PP2A, PP2B (also known as PP3), PP4, PP5, PP6, and PP7.

### The Structure of PP2A-Like Protein Phosphatases

Within the PPP family, PP2A, PP4, and PP6 come into being an independent cluster, whose catalytic subunits are most closely related, suggestive of a common origin (Chen et al., 2017). The catalytic subunits combine with scaffolding and/or regulatory subunits to form heterotrimeric or heterodimeric holoenzyme complexes (Brautigan and Shenolikar, 2018). Although their catalytic subunits have high sequence similarity, they have their own special structural compositions (Nasa and Kettenbach, 2020). PP2A is a heterotrimer holoenzyme complex consisting of a catalytic subunit, a scaffold subunit, and a regulatory subunit. The heterodimeric holoenzyme also named as core enzyme, composing of the catalytic and scaffold subunit, which is indispensable for the function of the holoenzyme (Price and Mumby, 2000). In higher eukaryotes, there are two isoforms in PP2A catalytic subunit (PPP2ACα and PPP2ACβ), which have 97% sequence similarity with each other. There are also two isoforms in PP2A scaffold subunit (PPP2R1α and PPP2R1β), which have abmost 87% sequence similarity (Saurin, 2018). The PP2A regulatory subunit has multiple members, which belong to four different families: B (B55), B′ (B56), B″ (B72), and B″′ (Striatin) family (Janssens and Goris, 2001). Therefore, the different combination of subunits results in various PP2A holoenzyme, differing in subcellular localization and distinct substrate specificity. For PP4, catalytic subunit combines with different regulatory subunits to form heterodimers or heterotrimers. The PP4 regulatory subunit has five isoforms: PPP4R1, PPP4R2, PPP4R3A, PPP4R3B, and PPP4R4 (Kloeker and Wadzinski, 1999; Cohen et al., 2005). Like other type 2A serine/threonine protein phosphatases, PP6 also works as a holoenzyme, consisting of a catalytic subunit, PPP6C, one of the three regulatory subunits including PPP6R1, PPP6R2 and PPP6R3, and one of the three ankyrin subunits including ARS-A, -B, -C (Stefansson and Brautigan, 2006; Stefansson et al., 2008).

### PP2A-Like Protein Phosphatases in Regulation of Meiotic Progression

#### PP2A

Among type 2A protein phosphatases, PP2A is the most famous and is widely researched. For a long time, accumulating evidence revealed its cellular and molecular importance. Studies also suggest that PP2A is involved in multiple steps of meiosis. In mouse oocytes, up-regulation of PP2A activity results in the meiotic arrest phenotype (Su et al., 2012). In *Oikopleura dioica*, PP2A is also necessary for meiotic arrest and precaution of parthenogenesis by restraining the abnormal Ca^2+^ burst (Matsuo et al., 2020). These results indicate that the function of PP2A is highly conserved in different organisms.

PP2A is essential for chromosome segregation during meiosis (Kerr et al., 2016). Several *in vitro* experiments have indicated that PP2A can associate with shugoshins and hold back the phosphorylation of Rec8 at the centromeres, a member of the cohesin complex, and finally stop split of Rec8 and keep the cohesion of chromatids in meiosis I (Kitajima et al., 2006; Lee et al., 2008; Rattani et al., 2013). In addition, Sororin and Shugoshin-PP2A collaborates in the regulation of centromeric cohesion during meiosis (Gomez et al., 2016). In Drosophila meiosis, the Shugoshin MEI-S332 and PP2A reciprocally promote localization of the other to centromeres and together they thus function to ensure accurate segregation (Pinto and Orr-Weaver, 2017). Also, a new study indicated that SCF (Skp1–Cul1–F box) -Fbxo42 down-regulates the protein level of the PP2A-B56 during synaptonemal complex assembly and maintenance (Barbosa et al., 2021). In mice spermatocytes, Previato de Almeida et al. found that Sgo2 is essential to protect centromere pairing by recruiting PP2A, while Sgo1 regulates non-exchange segregation by recruiting PP2A to centromeres (Previato de Almeida et al., 2019). In meiosis II, sister chromatids disjoin upon cleavage of centromeric Rec8. One assumption is that PP2A is separated from Rec8 by bipolar spindle forces in metaphase II. A recent experiment suggested that PP2A is removed from centromeres by the ubiquitin-ligase APC/C^Cdc20^, which can decrease the activity of Sgo1 and kinase Mps1 (Arguello-Miranda et al., 2017; Jonak et al., 2017).

Exact kinetochores-microtubule (KT-MT) is essential for correct chromosome segregation. In mitosis, correct KT-MT attachments are stabilized by inner sister KT stretching and the phosphorylation level of the KT. However, because of inherent property of the MI chromosomes, there is a difference between meiosis I and mitosis. This may explain the high incidence of KT-MT attachment errors in oocytes. In meiosis, PP2A-B56, which is regulated by the BubR1, is essential to determine the stability of KT-MT attachments independently of bivalent stretching (Yoshida et al., 2015). Overall, PP2A is targeted by Shugoshin and BubR1 to protect centromeric cohesion and stabilize KT-MT attachments in yeast and mouse meiosis. In *C. elegans* meiosis, the function of PP2A remains unclear. A recent study found that PP2A is necessary for female meiotic progression, such as spindle assembly and chromosome segregation. The mechanism is that BUB-1 targets PP2A-B′56 via a conserved LxxIxE motif and this regulation is necessary for correct meiotic progression (Bel Borja et al., 2020).

In addition, treatment with okadaic acid (OA) or calyculin-A (CL-A), which inhibits PP2A, caused an absence of microtubule polymerization and spindles. These studies have also showed that PP2A participated in microtubule organization and spindle formation (Lu et al., 2002). Protein phosphatase 2A regulatory subunit B55α (PP2A-B55α) is encoded by Ppp2r2a. Liang et al. found that PP2A-B55α was an important regulator of oocyte asymmetric division, chromosome congression, DNA damage response and spindle dynamics by RNA interference (Liang et al., 2017). In *Xenopus* oocytes, protein phosphatase 2A regulatory subunit B′56 (PP2A-B′56) and calcineurin (CaN) jointly contributes to APC/C^Cdc20^ activation by inhibiting phosphorylation of XErp1 (Heim et al., 2018). Two studies suggested that PP2A might be controlled by two distinct mechanisms in mouse oocytes. One is a post-translational modification by which MASTL (microtubule associated serine/threonine kinase-like), inhibit PP2A activity to promote anaphase (Adhikari et al., 2014). The other is CRL4-mediated degradation of the PP2A scaffold subunit, which reduces PP2A activity to facilitate non-reversible meiotic progression (Yu et al., 2015). These two regulation mechanisms of PP2A activity in conjunction with other meiotic regulators ensure precise meiotic progression in oocytes.

By using genetically modified mouse models, we further studied the functions of PP2A in oocyte meiosis We employed the conditional knockout method by using growth differentiation factor 9 (*Gdf9)-Cre* mice to gain mutant mice with depletion of PPP2R1α in oocytes in order to research its function in female meiosis. The results indicated that oocyte-specific depletion of PPP2R1α resulted in female subfertility because of production of aneuploid oocytes came from wrong separation of sister chromatids, but did not affect folliculogenesis, ovulation and spindle formation during meiosis II (Hu et al., 2014). Interestingly, another report generated conditional knockout mice by crossing *Ppp2ca*^f/f^ and (or) *Ppp2cb*^f/f^ with *Zp3-Cre* mice to study PP2A in female meiosis. They found that single knockout PPP2ACα females or PPP2ACβ females were fertile, indicating the paralogs were functionally redundant. Only the deficiency of both PPP2ACα and PPP2ACβ in oocytes finally resulted in female infertility (Tang et al., 2016). In this study, they also found that the PP2A can regulate chromosome behavior and bipolar spindle formation in meiosis I. PP2A counteracts Aurora kinase B/C to ensure bivalent stretching and KT-MT attachment stability (Tang et al., 2016). In contrast, PP2A is also essential for spermatogenesis, especially meiosis (Pan et al., 2015). However, the study is descriptive only, with a lack on mechanistic insight. It will be fascinating to reveal the deeply regulatory mechanism of PP2A in male meiosis.

#### PP4

PP4 has been widely studied over the past decade. However, there is a relative lack of information about PP4 in meiosis. In *C. elegans*, PP4 is indispensable for spindle formation during male meiosis, but it is not essential for female meiosis (Sumiyoshi et al., 2002). Moreover, at least four critical events in prophase require PP4, such as synapsis-independent chromosome pairing, prevention of non-homologous chromosome synapsis, DSB initiation, and crossover formation. The failure of these series of events eventually results in the failure of chiasmata formation (Sato-Carlton et al., 2014). In yeast, PP4 seems to be highly active during the whole meiotic progression. PP4 has an important role in single-end invasions, synaptonemal complex assembly, spindle formation and centromere pairing (Falk et al., 2010). To clarify whether PP4 has conserved functions in meiosis in mammalian species, we generated its catalytic subunit gene Ppp4c conditional knockout (*Ppp4c*^f/f^) mouse strain using CRISPR/Cas9 technology, and showed that loss of PPP4C did not affect male germ cell meiosis, acrosome formation, nuclear condensation and elongation, but caused the defect of cytoplasm removal, which in turn leads to the failure of spermiogenesis completion and male infertility (Han et al., 2020). Hence, the physiological roles and regulatory mechanism of PP4 in other organisms remain to be further studied.

#### PP6

Like other type 2A serine/threonine protein phosphatases, PP6 is also ubiquitously expressed in cells. However, PP6 has suffered less notice than its near relative PP2A and PP4, especially in meiosis. Until now, there only three papers about the functions of PP6 in meiosis. We showed that knockout of PP6 in oocytes from primary follicle stage resulted in female subfertility by disturbing MII spindle formation and MII exit after fertilization, indicating that PP6 can act as antagonizer to oocyte aneuploidy. But it is dispensable in oocyte meiotic maturation, follicle growth and ovulation (Hu et al., 2015). However, we showed that knockout of PP6 in oocytes from primordial follicle stage resulted in complete infertility of female mice. Deletion of PP6 caused meiotic prophase oocyte loss and abnormal folliculogenesis because of aberrant phosphorylation level of H2AX, which then led to lots of oocyte disappearance and eventually premature ovarian failure (POF). These results indicated that PP6 can also safeguard oocyte genomic integrity and regulate folliculogenesis during the long prophase I arrest (Hu et al., 2016). In male meiosis, our recent study by crossing *Ppp6c*^f/f^ mice with *Stra8-Cre* mice to obtain genetically mutant mice with specific malformation of the *Ppp6c* in male germ cells. We discovered that the mutant mice were male infertile and male germ cells were blocked at the pachytene stage during meiosis. Further study found that the loss of PP6 in male germ cells affected chromatin relaxation owing to abnormal MAPK pathway activity, thus stopping the recruitment of DSB repair factors to the appropriate sites on chromosomes (Lei et al., 2020).

### Perspectives

**FIGURE 1:**
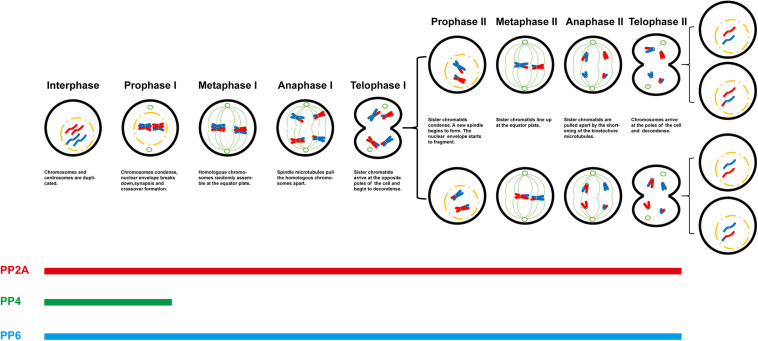
Role of PP2A-like protein phosphatases in meiotic progression.

Undoubtedly, protein phosphorylation is one of the most significant post-translational modifications during meiotic progression. The phosphorylation state of a special protein is regulated by protein kinases and protein phosphatases. As a member of PPP family, these new researches on PP2A-like protein phosphatases reported in past decades enriched the list of functions in meiosis, especially by employing conditional knockout mice (Table 1). Nonetheless, the most studies are descriptive only, with a lack of deep mechanistic insight. The special substrates of the different PP2A-like protein phosphatases are still a remaining impediment. In meiosis, it is not hard to notice that all three members can play the same role in special stages or special biological events (Figure 1). Are they functionally redundant? In the future, these unanswered questions remain to be further studied. Quantitative mass spectrometry-based proteomic and phosphoprotoemic approaches maybe provide a solution for understanding regulatory functional mechanism of PP2A-like protein phosphatases in meiotic progress. In addition, the progress of short linear motifs (SLiM) also provides a method to study their biological functions and distinct substrates. These will fill the gaps in the regulation networks of phosphorylation.

**TABLE 1:** Consequences of deletions of PP2A-like protein phosphatases in mouse germ cells.

PP2A-like protein phosphatases	Subunit deleted	Cre recombinase	Phenotype	References
PP2A	PPP2R1α	Gdf9	Subfertile	Hu et al., 2014
	PPP2ACα	Zp3	Fertile	Tang et al., 2016
	PPP2ACβ	Zp3	Fertile	Tang et al., 2016
	PPP2ACα& PPP2ACβ	Zp3	Infertile	Tang et al., 2016
	PPP2ACα	DDx4	Infertile	Pan et al., 2015
PP4	PPP4C	Stra8	Infertile	Han et al., 2020
PP6	PPP6C	Zp3	Subfertile	Hu et al., 2015
	PPP6C	Gdf9	Infertile	Hu et al., 2016
	PPP6C	Stra8	Infertile	Lei et al., 2020

## Author Contributions

W-LL collected the data, drew the picture and tables, and wrote the manuscript. W-PQ and Q-YS revised the manuscript. All authors contributed to the article and approved the submitted version.

## Conflict of Interest

The authors declare that the research was conducted in the absence of any commercial or financial relationships that could be construed as a potential conflict of interest.

## References

[B1] Adhikari D. Diril M. K. Busayavalasa K. Risal S. Nakagawa S. Lindkvist R. (2014). Mastl is required for timely activation of APC/C in meiosis I and Cdk1 reactivation in meiosis II. *J. Cell Biol.* 206 843–853. 10.1083/jcb.201406033 25246615 PMC4178961

[B2] Afshar K. Werner M. E. Tse Y. C. Glotzer M. Gönczy P. (2016). Regulation of cortical contractility and spindle positioning by the protein phosphatase 6 PPH-6 in one-cell stage C. elegans embryos. *Development* 143:2689. 10.1242/dev.141515 27436042 PMC4958344

[B3] Arguello-Miranda O. Zagoriy I. Mengoli V. Rojas J. Jonak K. Oz T. (2017). Casein Kinase 1 Coordinates Cohesin Cleavage. Gametogenesis, and Exit from M Phase in Meiosis II. *Dev. Cell* 40 37–52. 10.1016/j.devcel.2016.11.021 28017619

[B4] Barbosa P. Zhaunova L. Debilio S. Steccanella V. Kelly V. Ly T. (2021). SCF-Fbxo42 promotes synaptonemal complex assembly by downregulating PP2A-B56. *J. Cell Biol.* 220:202009167. 10.1083/jcb.202009167 33382409 PMC7780726

[B5] Bel Borja L. Soubigou F. Taylor S. J. P. Fraguas Bringas C. Budrewicz J. Lara-Gonzalez P. (2020). BUB-1 targets PP2A:B56 to regulate chromosome congression during meiosis I in C. elegans oocytes. *eLife* 9:65307. 10.7554/eLife.65307 PMC7787666 33355089

[B6] Berchowitz L. E. Copenhaver G. P. (2010). Genetic interference: don’t stand so close to me. *Curr. Genomics* 11 91–102. 10.2174/138920210790886835 20885817 PMC2874225

[B7] Bielinski V. A. Mumby M. C. (2007). Functional analysis of the PP2A subfamily of protein phosphatases in regulating Drosophila S6 kinase. *Exp. Cell Res.* 313 3117–3126. 10.1016/j.yexcr.2007.05.008 17570358 PMC1991331

[B8] Bishop D. K. Zickler D. (2004). Early decision; meiotic crossover interference prior to stable strand exchange and synapsis. *Cell* 117 9–15. 10.1016/s0092-8674(04)00297-1 15066278

[B9] Bornslaeger E. A. Mattei P. Schultz R. M. (1986). Involvement of cAMP-dependent protein kinase and protein phosphorylation in regulation of mouse oocyte maturation. *Dev. Biol.* 114 453–462. 10.1016/0012-1606(86)90209-5 2420661

[B10] Brautigan D. L. (2013). Protein Ser/Thr phosphatases–the ugly ducklings of cell signalling. *Febs J.* 280 324–345. 10.1111/j.1742-4658.2012.08609.x 22519956

[B11] Brautigan D. L. Shenolikar S. (2018). Protein Serine/Threonine Phosphatases: Keys to Unlocking Regulators and Substrates. *Annu Rev Biochem* 87 921–964. 10.1146/annurev-biochem-062917-012332 29925267

[B12] Canela N. Rodriguez-Vilarrupla A. Estanyol J. M. Diaz C. Pujol M. J. Agell N. (2003). The SET protein regulates G2/M transition by modulating cyclin B-cyclin-dependent kinase 1 activity. *J. Biol. Chem.* 278 1158–1164. 10.1074/jbc.M207497200 12407107

[B13] Cassimeris L. (1999). Accessory protein regulation of microtubule dynamics throughout the cell cycle. *Curr. Opin. Cell Biol.* 11 134–141. 10.1016/s0955-0674(99)80017-9 10047516

[B14] Chen M. J. Dixon J. E. Manning G. (2017). Genomics and evolution of protein phosphatases. *Sci. Signal.* 10:aag1796. 10.1126/scisignal.aag1796 28400531

[B15] Cohen P. T. Philp A. Vazquez-Martin C. (2005). Protein phosphatase 4–from obscurity to vital functions. *FEBS Lett.* 579 3278–3286. 10.1016/j.febslet.2005.04.070 15913612

[B16] Falk J. E. Chan A. C. Hoffmann E. Hochwagen A. (2010). A Mec1- and PP4-dependent checkpoint couples centromere pairing to meiotic recombination. *Dev. Cell* 19 599–611. 10.1016/j.devcel.2010.09.006 20951350

[B17] Gomez R. Felipe-Medina N. Ruiz-Torres M. Berenguer I. Viera A. Perez S. (2016). Sororin loads to the synaptonemal complex central region independently of meiotic cohesin complexes. *EMBO Rep.* 17 695–707. 10.15252/embr.201541060 26951638 PMC5341523

[B18] Han F. Dong M. Z. Lei W. L. Xu Z. L. Gao F. Schatten H. (2020). Oligoasthenoteratospermia and sperm tail bending in PPP4C-deficient mice. *Mol. Hum. Reprod* 2020:gaaa083. 10.1093/molehr/gaaa083 33543287

[B19] Heim A. Tischer T. Mayer T. U. (2018). Calcineurin promotes APC/C activation at meiotic exit by acting on both XErp1 and Cdc20. *EMBO Rep.* 19:201846433. 10.15252/embr.201846433 30373936 PMC6280790

[B20] Hu M. W. Meng T. G. Jiang Z. Z. Dong M. Z. Schatten H. Xu X. (2016). Protein Phosphatase 6 Protects Prophase I-Arrested Oocytes by Safeguarding Genomic Integrity. *PLoS Genet.* 12:e1006513. 10.1371/journal.pgen.1006513 27930667 PMC5179128

[B21] Hu M. W. Wang Z. B. Jiang Z. Z. Qi S. T. Huang L. Liang Q. X. (2014). Scaffold subunit Aalpha of PP2A is essential for female meiosis and fertility in mice. *Biol. Reprod* 91:19. 10.1095/biolreprod.114.120220 24899574

[B22] Hu M. W. Wang Z. B. Teng Y. Jiang Z. Z. Ma X. S. Hou N. (2015). Loss of protein phosphatase 6 in oocytes causes failure of meiosis II exit and impaired female fertility. *J. Cell Sci.* 128 3769–3780. 10.1242/jcs.173179 26349807

[B23] Hunter T. (1995). Protein kinases and phosphatases: the yin and yang of protein phosphorylation and signaling. *Cell* 80 225–236. 10.1016/0092-8674(95)90405-0 7834742

[B24] Hunter T. (2014). The genesis of tyrosine phosphorylation. *Cold Spring Harb. Perspect. Biol.* 6:a020644. 10.1101/cshperspect.a020644 24789824 PMC3996475

[B25] Janssens V. Goris J. (2001). Protein phosphatase 2A: a highly regulated family of serine/threonine phosphatases implicated in cell growth and signalling. *Biochem J.* 353(Pt 3), 417–439. 10.1042/0264-6021:3530417 11171037 PMC1221586

[B26] Jonak K. Zagoriy I. Oz T. Graf P. Rojas J. Mengoli V. (2017). APC/C-Cdc20 mediates deprotection of centromeric cohesin at meiosis II in yeast. *Cell Cycle* 16 1145–1152. 10.1080/15384101.2017.1320628 28514186 PMC5499901

[B27] Keating L. Touati S. A. Wassmann K. (2020). A PP2A-B56-Centered View on Metaphase-to-Anaphase Transition in Mouse Oocyte Meiosis I. *Cells* 9:9020390. 10.3390/cells9020390 32046180 PMC7072534

[B28] Kerk D. Templeton G. Moorhead G. B. (2008). Evolutionary radiation pattern of novel protein phosphatases revealed by analysis of protein data from the completely sequenced genomes of humans, green algae, and higher plants. *Plant Physiol.* 146 351–367. 10.1104/pp.107.111393 18156295 PMC2245839

[B29] Kerr G. W. Wong J. H. Arumugam P. (2016). PP2A(Cdc55)’s role in reductional chromosome segregation during achiasmate meiosis in budding yeast is independent of its FEAR function. *Sci. Rep.* 6:30397. 10.1038/srep30397 27455870 PMC4960654

[B30] Kitajima T. S. Sakuno T. Ishiguro K. Iemura S. Natsume T. Kawashima S. A. (2006). Shugoshin collaborates with protein phosphatase 2A to protect cohesin. *Nature* 441 46–52. 10.1038/nature04663 16541025

[B31] Kloeker S. Wadzinski B. E. (1999). Purification and identification of a novel subunit of protein serine/threonine phosphatase 4. *J. Biol. Chem.* 274 5339–5347. 10.1074/jbc.274.9.5339 10026142

[B32] Lad C. Williams N. H. Wolfenden R. (2003). The rate of hydrolysis of phosphomonoester dianions and the exceptional catalytic proficiencies of protein and inositol phosphatases. *Proc. Natl. Acad. Sci. U S A* 100 5607–5610. 10.1073/pnas.0631607100 12721374 PMC156248

[B33] Lee J. Kitajima T. S. Tanno Y. Yoshida K. Morita T. Miyano T. (2008). Unified mode of centromeric protection by shugoshin in mammalian oocytes and somatic cells. *Nat Cell Biol* 10 42–52. 10.1038/ncb1667 18084284

[B34] Lei W. L. Han F. Hu M. W. Liang Q. X. Meng T. G. Zhou Q. (2020). Protein phosphatase 6 is a key factor regulating spermatogenesis. *Cell Death Differ* 27 1952–1964. 10.1038/s41418-019-0472-9 31819157 PMC7244475

[B35] Liang S. Guo J. Choi J. W. Shin K. T. Wang H. Y. Jo Y. J. (2017). Protein phosphatase 2A regulatory subunit B55alpha functions in mouse oocyte maturation and early embryonic development. *Oncotarget* 8 26979–26991. 10.18632/oncotarget.15927 28439046 PMC5432312

[B36] Lillo C. Kataya A. R. Heidari B. Creighton M. T. Nemie-Feyissa D. Ginbot Z. (2014). Protein phosphatases PP2A, PP4 and PP6: mediators and regulators in development and responses to environmental cues. *Plant. Cell Environ.* 37 2631–2648. 10.1111/pce.12364 24810976

[B37] Lu Q. Dunn R. L. Angeles R. Smith G. D. (2002). Regulation of spindle formation by active mitogen-activated protein kinase and protein phosphatase 2A during mouse oocyte meiosis. *Biol. Reprod.* 66 29–37. 10.1095/biolreprod66.1.29 11751260

[B38] Lu Q. Smith G. D. Chen D. Y. Yang Z. Han Z. M. Schatten H. (2001). Phosphorylation of mitogen-activated protein kinase is regulated by protein kinase C, cyclic 3′,5′-adenosine monophosphate, and protein phosphatase modulators during meiosis resumption in rat oocytes. *Biol. Reprod* 64 1444–1450. 10.1095/biolreprod64.5.1444 11319150

[B39] Matsuo M. Onuma T. A. Omotezako T. Nishida H. (2020). Protein phosphatase 2A is essential to maintain meiotic arrest, and to prevent Ca(2+) burst at spawning and eventual parthenogenesis in the larvacean Oikopleura dioica. *Dev. Biol.* 460 155–163. 10.1016/j.ydbio.2019.12.005 31857067

[B40] Mumby M. C. Walter G. (1993). Protein serine/threonine phosphatases: structure, regulation, and functions in cell growth. *Physiol. Rev.* 73 673–699. 10.1152/physrev.1993.73.4.673 8415923

[B41] Nasa I. Kettenbach A. N. (2020). Effects of carboxyl-terminal methylation on holoenzyme function of the PP2A subfamily. *Biochem. Soc. Transac.* 48 2015–2027. 10.1042/bst20200177 33125487 PMC8380034

[B42] Nilsson J. (2019). Protein phosphatases in the regulation of mitosis. *J. Cell Biol.* 218 395–409. 10.1083/jcb.201809138 30446607 PMC6363451

[B43] Pan X. Chen X. Tong X. Tang C. Li J. (2015). Ppp2ca knockout in mice spermatogenesis. *Reproduction* 149 385–391. 10.1530/REP-14-0231 25628439

[B44] Pearlman S. M. Serber Z. Ferrell J. E. Jr. (2011). A mechanism for the evolution of phosphorylation sites. *Cell* 147 934–946. 10.1016/j.cell.2011.08.052 22078888 PMC3220604

[B45] Pinto B. S. Orr-Weaver T. L. (2017). Drosophila protein phosphatases 2A B’ Wdb and Wrd regulate meiotic centromere localization and function of the MEI-S332 Shugoshin. *Proc. Natl. Acad. Sci. U S A* 114 12988–12993. 10.1073/pnas.1718450114 29158400 PMC5724294

[B46] Previato de Almeida L. Evatt J. M. Chuong H. H. Kurdzo E. L. Eyster C. A. (2019). Shugoshin protects centromere pairing and promotes segregation of nonexchange partner chromosomes in meiosis. *Proc. Natl. Acad. Sci. U S A* 116 9417–9422. 10.1073/pnas.1902526116 31019073 PMC6511000

[B47] Price N. E. Mumby M. C. (2000). Effects of regulatory subunits on the kinetics of protein phosphatase 2A. *Biochemistry* 39 11312–11318. 10.1021/bi0008478 10985776

[B48] Qi S. T. Wang Z. B. Ouyang Y. C. Zhang Q. H. Hu M. W. Huang X. (2013). Overexpression of SETbeta, a protein localizing to centromeres, causes precocious separation of chromatids during the first meiosis of mouse oocytes. *J. Cell Sci.* 126(Pt 7), 1595–1603. 10.1242/jcs.116541 23444375

[B49] Rattani A. Wolna M. Ploquin M. Helmhart W. Morrone S. Mayer B. (2013). Sgol2 provides a regulatory platform that coordinates essential cell cycle processes during meiosis I in oocytes. *Elife* 2:e01133. 10.7554/eLife.01133 24192037 PMC3816256

[B50] Sato-Carlton A. Li X. Crawley O. Testori S. Martinez-Perez E. Sugimoto A. (2014). Protein phosphatase 4 promotes chromosome pairing and synapsis, and contributes to maintaining crossover competence with increasing age. *PLoS Genet.* 10:e1004638. 10.1371/journal.pgen.1004638 25340746 PMC4207613

[B51] Saurin A. T. (2018). Kinase and Phosphatase Cross-Talk at the Kinetochore. *Front. Cell Dev. Biol.* 6:62. 10.3389/fcell.2018.00062 29971233 PMC6018199

[B52] Stefansson B. Brautigan D. L. (2006). Protein phosphatase 6 subunit with conserved Sit4-associated protein domain targets IkappaBepsilon. *J. Biol. Chem.* 281 22624–22634. 10.1074/jbc.M601772200 16769727

[B53] Stefansson B. Ohama T. Daugherty A. E. Brautigan D. L. (2008). Protein phosphatase 6 regulatory subunits composed of ankyrin repeat domains. *Biochemistry* 47 1442–1451. 10.1021/bi7022877 18186651

[B54] Su Y. Q. Sugiura K. Sun F. Pendola J. K. Cox G. A. Handel M. A. (2012). MARF1 regulates essential oogenic processes in mice. *Science* 335 1496–1499. 10.1126/science.1214680 22442484 PMC3612990

[B55] Subramani S. Jayapalan S. Kalpana R. Natarajan J. (2013). HomoKinase: A Curated Database of Human Protein Kinases. *ISRN Computational. Biol.* 2013:417634. 10.1155/2013/417634

[B56] Sumiyoshi E. Sugimoto A. Yamamoto M. (2002). Protein phosphatase 4 is required for centrosome maturation in mitosis and sperm meiosis in C. elegans. *J. Cell Sci.* 115(Pt 7), 1403–1410. 11896188 10.1242/jcs.115.7.1403

[B57] Tang A. Shi P. Song A. Zou D. Zhou Y. Gu P. (2016). PP2A regulates kinetochore-microtubule attachment during meiosis I in oocyte. *Cell Cycle* 15 1450–1461. 10.1080/15384101.2016.1175256 27096707 PMC4934050

[B58] Wolgemuth D. J. Roberts S. S. (2010). Regulating mitosis and meiosis in the male germ line: critical functions for cyclins. *Philos. Trans. R. Soc. Lond. B Biol. Sci.* 365 1653–1662. 10.1098/rstb.2009.0254 20403876 PMC2871921

[B59] Yoshida S. Kaido M. Kitajima T. S. (2015). Inherent Instability of Correct Kinetochore-Microtubule Attachments during Meiosis I in Oocytes. *Dev. Cell* 33 589–602. 10.1016/j.devcel.2015.04.020 26028219

[B60] Yu C. Ji S. Y. Sha Q. Q. Sun Q. Y. Fan H. Y. (2015). CRL4-DCAF1 ubiquitin E3 ligase directs protein phosphatase 2A degradation to control oocyte meiotic maturation. *Nat. Commun.* 6:8017. 10.1038/ncomms9017 26281983 PMC4557334

